# A rare-earth free magnesium alloy with improved intrinsic ductility

**DOI:** 10.1038/s41598-017-10384-0

**Published:** 2017-09-05

**Authors:** S. Sandlöbes, M. Friák, S. Korte-Kerzel, Z. Pei, J. Neugebauer, D. Raabe

**Affiliations:** 1 Institut für Metallkunde und Metallphysik, Kopernikusstr. 14, RWTH Aachen University, 52074 Aachen, Germany; 2 Institute of Physics of Materials, Academy of Sciences of the Czech Republic, v.v.i., Žižkova 22, Brno, 616 62 Czech Republic; 3 Max-Planck-Institut für Eisenforschung GmbH, Max-Planck-Straβe 1, 40237 Düsseldorf, Germany

## Abstract

Metals are the backbone of manufacturing owing to their strength and formability. Compared to polymers they have high mass density. There is, however, one exception: magnesium. It has a density of only 1.7 g/cm^3^, making it the lightest structural material, 4.5 times lighter than steels, 1.7 times lighter than aluminum, and even slightly lighter than carbon fibers. Yet, the widespread use of magnesium is hampered by its intrinsic brittleness. While other metallic alloys have multiple dislocation slip systems, enabling their well-known ductility, the hexagonal lattice of magnesium offers insufficient modes of deformation, rendering it intrinsically brittle. We have developed a quantum-mechanically derived treasure map which screens solid solution combinations with electronic bonding, structure and volume descriptors for similarity to the ductile magnesium-rare earth alloys. Using this insight we synthesized a surprisingly simple, compositionally lean, low-cost and industry-compatible new alloy which is over 4 times more ductile and 40% stronger than pure magnesium. The alloy contains 1 wt.% aluminum and 0.1 wt.% calcium, two inexpensive elements which are compatible with downstream recycling constraints.

## Introduction

Permanent shape changes of metals are enabled by the motion of line defects that break atomic bonds and create new ones along densely packed lattice directions. These defects are referred to as dislocations and their crystallographic features as slip systems. Each dislocation shears the material by one atomic spacing. The motion of up to one light year of dislocation length per cubic meter (10^16^ m/m^3^) thus enables macroscopic deformation and forming.

Metals are typically used in polycrystalline form with crystal sizes of up to several micrometers. A square meter of auto skin sheet for instance can consist of more than 10^10^ crystals. Since individual crystals deform only along specific crystallographic directions, macroscopic shape changes require deformation compatibility among them. Accommodating arbitrary deformations thus requires at least 5 independent deformation systems in each crystal to be active^1^. While cubic metallic crystals (e.g. steels, Al-alloys) have a sufficiently high number of independent deformation systems due to their high crystal symmetry, most hexagonal crystals exhibit insufficient independent deformation systems^2, 3^.

**Figure 1:**
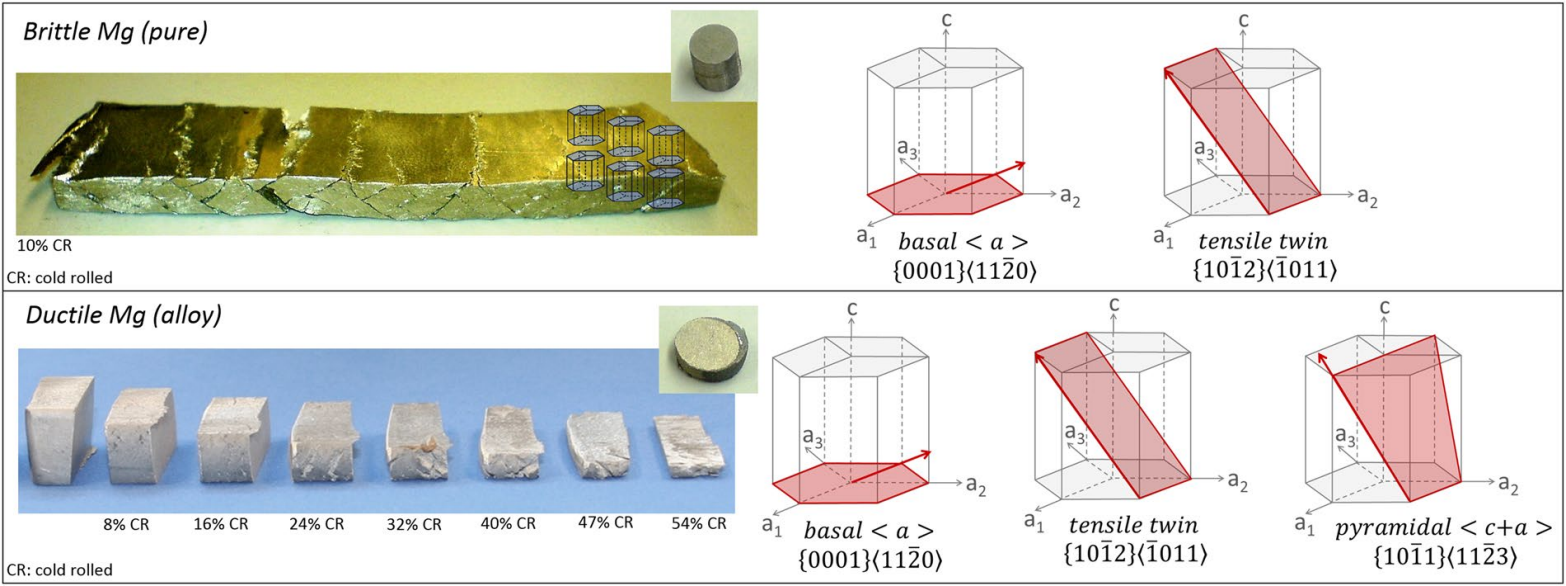
Pure polycrystalline magnesium failing in a brittle manner already at low deformations, shown here in the rolled state. Pure Mg fractured along macroscopic shear bands when cold rolled to 10% thickness reduction whereas Mg-1Al-0.1Ca could be cold rolled to 54% thickness reduction in several rolling passes of 8% thickness reduction per rolling pass; small sheet sections cut from the rolled sheet after each consecutive rolling pass are presented. Early failure of pure Mg occurs due to the restriction to mainly basal <a> dislocation slip and tensile twinning. Additional activation of pyramidal <c + a> dislocation slip enables improved room temperature formability specimens of the new Mg-Al-Ca alloy before and after deformation. The corresponding tensile stress-strain curves are shown in Fig. 3 (pure Mg blue line and Mg-1Al-0.1Ca in red).

Magnesium and its alloys, as the lightest class of structural metals, have a hexagonal lattice structure. Thus, despite some excellent properties such as low mass density, good castability and efficient recyclability^4^, their wider industrial application is fundamentally impeded by their intrinsic poor room temperature ductility. The lack of room temperature formability is caused by deformation being governed by $\{0001\}\langle 11\bar{2}0\rangle$ basal <a> dislocation slip and $\{10\bar{1}2\}\langle 10\bar{1}\bar{1}\rangle$ tensile twinning^2, 3, 5–14^. Basal <a> slip does not allow accommodation of strain along the crystal c-axis but rather a rotation of the crystal c-axes parallel to the loading direction^2, 9, 14^, leading to the so-called basal texture component^9^. Consequently, magnesium fails at low strains, Fig. 1.

Strain along the crystal c-axis can only be accommodated by the activation of non-basal slip in hexagonal crystals, i.e. it is crucial for compatible polycrystalline deformation of Mg^2, 6, 12–16^. Even if dislocations with a <c + a> Burgers vector are formed they do not prevail in pure Mg: Curtin *et al*.^15^ recently showed by using molecular dynamics simulations that dislocations with non-basal Burgers vectors are not stable but dissociate and relax back onto the basal plane in pure Mg.

Manipulation of the activation and stability of dislocations and slip systems is possible through the addition of alloying elements. Specifically, dilute alloying with yttrium and rare earth (RE) elements has been shown to improve the room temperature ductility significantly^4–13^. Recent studies by combined transmission electron microscopy (TEM) measurements and *ab initio* calculations revealed that such a ductility increase in Mg-Y and Mg-RE alloys is caused by an increased activity of <c + a> dislocation slip providing out-of-basal-plane shear^6–8, 11–13^. Such an intrinsic change of the activation of dislocation slip systems worked so far only for yttrium, rare earth elements^12, 13, 17^ and lithium in solid solution^11, 18, 19^ rendering such alloys expensive, difficult to process and incompatible with recycling constraints.

An alternative, processing-oriented option of solving the brittleness problem lies in breaking these prevalent basal textures up into non-basal texture components which enable ductile deformation of polycrystalline compounds without the activation of pyramidal slip. The concepts which currently exist aim at ductilizing commercial magnesium alloys by employing either texture or grain size engineering through laborious and expensive processing technologies [e.g. refs 4, 20–28] such as extrusion [e.g. refs 21–23] or asymmetric forming [e.g. refs 25, 26], severe plastic deformation to produce ultra-fine grained microstructures [e.g. refs 24, 27], or the precipitation of second phases through higher alloying additions [e.g. refs 29–31]. All these concepts, although improving ductility, do not aim at improving the intrinsic brittleness of magnesium but rather delay failure through smart, yet expensive processing or extensive alloying.

We follow an alternative approach aiming at improving the fundamental plasticity mechanisms that control intrinsic ductility, i.e. the intrinsic activation of multiple deformation systems. Our preliminary studies have revealed that the facilitated activation of out-of-basal-plane shear modes through the addition of Y or RE elements to Mg is correlated with a significantly decreased I_1_ intrinsic stacking fault energy (I_1_ SFE) (The I_1_ intrinsic stacking fault changes the stacking sequence from …ABABAB… to … ABABCBCB …)^12, 13^. In these studies the I_1_ SFE has been found to decrease with increasing Y and RE concentration. We propose that this reduction of the I_1_ SFE can be used as a guiding parameter (among others) connected with the ductility increase in the Mg-Y and Mg-RE systems acting as follows: The enhanced ductility is enabled by an increased activity of pyramidal <c + a> dislocations as slip modes out of the basal plane. It is the nucleation of <c + a> dislocations which forms the critical step in providing out-of-basal-plane shear. This is associated with the I_1_ SF: the sessile I_1_ SF, whose energy decreases with Y or RE alloying, is bound by a pyramidal partial dislocation. This dislocation arrangement enables the formation of dislocation structures on pyramidal planes^12, 13, 32, 33^, i.e. it acts as heterogeneous nucleation source for pyramidal <c + a> dislocations.

Based on these studies we conducted *ab initio* assessments of the fundamental thermodynamic, energetic (elastic energy) and structural-volumetric interactions of yttrium and RE atoms in Mg in solid solution and their effects on the I_1_ SFE to better understand the origin of slip system selection in these ductile model alloys. Considering only 60 commercially used elements as solute ingredients, i.e. >10^120^ possible alloy variants, shows that an empirical approach for identifying Mg solid solution alloys with properties similar to those of magnesium-rare earth alloys is hopeless.

Hence, we developed a quantum mechanically guided treasure map for Mg alloying^17^. The key idea of this concept is to start from a Mg-Y alloy that has the desired ductility, yet, is commercially not attractive, and search for alternative alloy compositions which match the reference system as closely as possible for selected (and easy to obtain) element-specific properties. This proximity between two alloys is expressed by a similarity index

1 $${Y}_{c}=1-{\{\sum _{\alpha }{w}_{\alpha }{(\alpha -{\alpha }_{c})}^{2}\}}^{1/2}$$

where the subscript c describes the chemical composition of the new alloy, *α* describes a selected set of element-specific properties and ${w}_{\alpha }$ are the respective weighting factors. This proximity factor ${Y}_{c}\,\,$ is referred to as yttrium-similarity index, YSI, with values closer to 1 indicating a higher similarity to Y.

To determine a suitable set of elemental properties and weighting factors we used density-functional theory to compute reference quantities for an extensive set of solid-solution binary Mg_1−x_X_x_ alloys (x ≪ 1)^17^. By correlation analysis on this extensive data set we identified three specific, strong fundamental property correlations: the atomic volume of pure solutes, their electronegativity and their bulk modulus^17^. From the correlation coefficients we obtained the weights ${w}_{\alpha }$.

**Figure 2:**
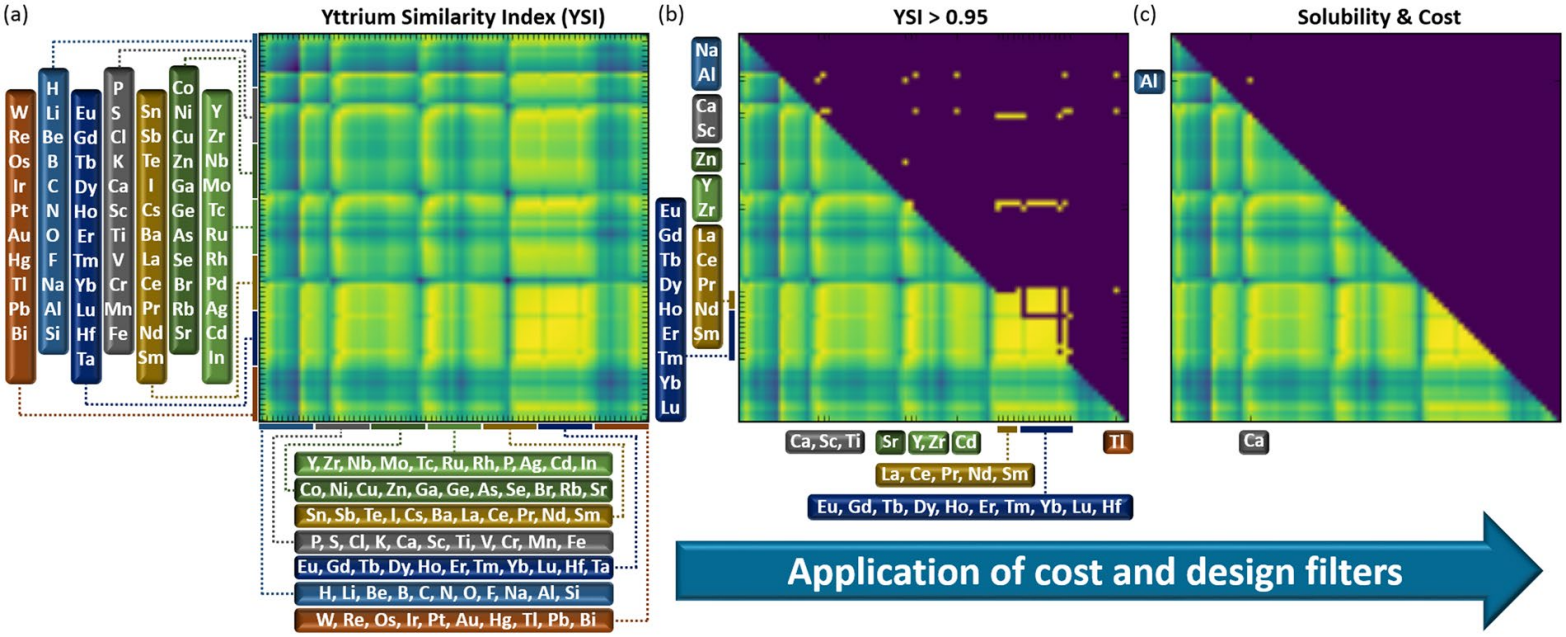
Computed values of the yttrium-similarity index, YSI (Eq. 1) for the 2850 solute pairs computed in this study and visualized in the form of a symmetric matrix (**a**) with yellow indicating a high similarity and blue a low one. Solute pairs that have a high index (YSI > 0.95) are shown in the upper triangular part in (**b**). Applying a cost and solubility filter (see text) only a single pair, Al-Ca, remains (**c**).

Specifically, we screened 2850 ternary combinations (Fig. 2a)^17^ and identified 17 promising ternary alloys highlighted in Fig. 2(b) with YSI values ≥ 0.95. Figure 2a shows a symmetric matrix of all 2850 solute pairs computed, where the respective solutes are given on the x- and y-axes. The intersection points of each solute pair of the y- and x-axis are marked by a colored point indicating the similarity of that pair to Y. Yellow color corresponds to a high similarity to Y (high YSI) and blue color to a low YSI. In the upper triangle of Fig. 2b only those solute pairs which were calculated to have an YSI above 0.95 (i.e. 95% or higher similarity to Y) are shown. The so identified solutes are listed at the x- and y-axis in Fig. 2b. Screening this list of predicted alloys reveals that most of them contain rare earth elements. Yet, 11 of them are non-RE/Y containing solute pairs, namely, Ti-Ca; Cd-Na; Sr-Al; Ca-Al; Tl-Ca; Cd-Ca; Sr-Zn; Hf-Ca; Zr-Na; Tl-Na; Zr-Ca. A full list of all computed YSI values for all considered solute pairs is given in ref. 17. After imposing first this non-RE/Y filter we have applied a second selection filter ruling out those of the remaining solute pairs which are incompatible with recycling constraints (Cd, Zr, Hf, Tl), toxic (Cd), not sufficiently soluble (Zr, Hf) in Mg or too expensive (Zr, Tl, Hf, Sr). After applying this final filter, only one alloy system remains, as displayed by the yellow point in Fig. 2c, viz. Mg-Al-Ca. The thus identified ternary Mg-Al-Ca alloy is fully compatible with commercial metallurgy and contains two inexpensive and non-toxic elements. Interestingly, ternary Mg alloys containing Ca and Al have of course been synthesized before, however, with higher alloying contents, above the solubility limit of Ca [e.g. refs 34 and 35], and showing no beneficial mechanical properties. Since the starting point of our similarity approach is a homogeneous, precipitate free Mg-Y solid solution alloy, we carefully checked that the doping levels of Al and Ca are below the solubility limit in contrast to previous work on Mg-Al-Ca alloys.

**Figure 3:**
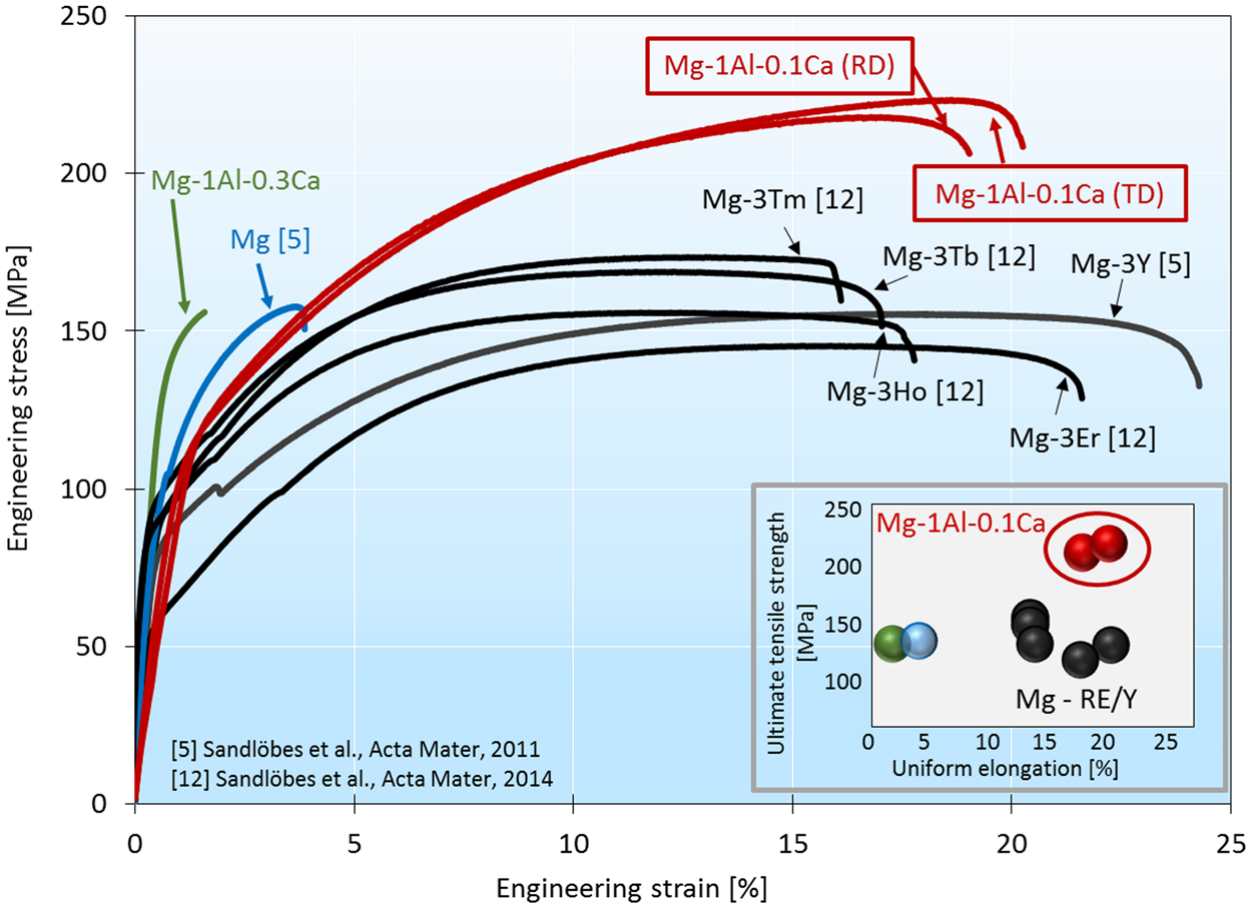
Engineering stress-strain curves of the new Mg-Al-Ca alloy shown in Fig. 1 in comparison with not engineered (other than simple homogenization treatment) solid solution Mg-Y, Mg-RE, pure Mg and Mg-Al-0.3Ca^6, 11^. The inset shows the ultimate tensile strength – uniform elongation diagram of the same alloys displaying the superior mechanical properties of the new alloy. RD: rolling direction; TD: transverse direction. Compositions are in weight %.

Following this *ab initio* guided approach, we identified and synthesized a new material in the Mg-Al-Ca system, namely, Mg-1Al-0.1Ca (wt.%). Figure 3 shows the tensile stress-strain behavior of the new as-homogenized (as-cast; 50% hot rolled at 430 °C; recrystallization annealing at 450 °C for 15 min; water quenching) Mg-1Al-0.1Ca alloy in direct comparison with pure Mg and binary solid solution Mg-RE and Mg-Y alloys^6, 13^ revealing superior mechanical properties with a tensile elongation of about 20%, i.e. 4 times more ductile than pure magnesium, well-balanced constant work hardening, and an ultimate tensile strength of about 220 MPa, exceeding that of pure Mg by 40%. Figure 3 shows a comparison with other solid solution alloys which do not contain any second phase precipitates (despite our less dilute Mg-1Al-0.3Ca alloy) and have not been processed to obtain texture weakening or grain refinement.

To show the importance of staying below the solubility limit as discussed above, we tested another, less-dilute Mg-Ca-Al alloy, i.e. Mg-1Al-0.3Ca (wt.%). This alloy with a Ca content slightly above the solubility limit shows brittle failure (see Fig. 3) which is related to the formation of hard and brittle Mg_2_Ca Laves phase precipitates. Hence, the mechanisms of early failure of the Mg-1Al-0.3Ca alloy are different from those in pure Mg. In the Mg-1Al-0.3Ca alloy Laves phase precipitates have formed at grain boundaries. The brittle failure is then not caused by strain localization, as in pure Mg, but due to the presence of the hard Laves phase precipitates which act as microstructurally weak points. Similar ductilization trends were also very recently observed by Zeng *et al*.^36^ and Suh *et al*.^37^ for Mg-Zn-Ca alloys (actually, our analysis predicts an yttrium similarity index of 0.942 for Zn-Ca in case of equal concentration of both solutes) and also by Chino *et al*. and Nakata *et al*.^22, 23, 38^ who showed ductility increase through texture weakening in Mg-Zn-Ca and Mg-Al-Ca based alloys^22, 23^ and through grain refinement and basal texture weakening due to severe plastic deformation^38^. Enhanced room temperature ductility through the activation of <c + a> slip has been also reported for Mg-Li alloys^11, 18, 19^ (our analysis predicts an yttrium similarity index of 0.837 for binary Mg-Li alloys). Considering the price aspect, Zn is 2-3.5 times and Li 3-3.5 times more expensive than Al which we combined with Ca in our alloy.

Zn is one of the most important alloying elements in Mg alloys, however, when alloyed in the form of a binary Mg-Zn alloy, the material possesses only a low calculated YSI of 0.743 and only one solute pair which contains Zn has a calculated YSI of >0.95: Sr-Zn. Alloying of Zn has been reported to have various effects on Mg, namely, precipitation strengthening (e.g. AZ alloys^39, 40^), hardening of basal <a> dislocation slip and softening of prismatic <a> dislocation slip^41–43^ and grain refinement^39, 40, 44, 45^. Further, Zn has been reported to moderately increase the ductility of Mg, however, not as substantial as reported for Y/RE additions and without pronounced activity of <c + a> dislocation slip^39, 44, 45^.

When considering commercial Al and/or Zn containing alloys such as AZ31, AM60, AZ61 and AZ91 a broad range of mechanical properties has been reported^21, 24, 46–62^, where high strength of up to about 300 MPa^62^ and/or high ductility of up to 40%^24^ have been observed mainly for extruded or SPD treated alloys with UFG grain size and/or weakened basal texture^21, 24, 46–62^, but limited ductility for coarser grained alloys with basal-type textures^24, 47, 54, 63^. Particular work has been performed on increasing the formability of these alloys through novel and advanced processing routes^21, 24, 46–62^. Yamashita *et al*.^47^ have studied an extruded binary Mg-0.9Al (wt.-%) alloy before and after severe plastic deformation processing. They have reported a yield stress of about 55 MPa and tensile elongation of about 3% for the as-extruded binary Mg-0.9Al which could subsequently be increased to a yield stress of about 150 MPa and tensile elongation of about 17.5% via the ensuing severe plastic deformation causing substantial grain refinement and texture weakening^47^.

**Figure 4:**
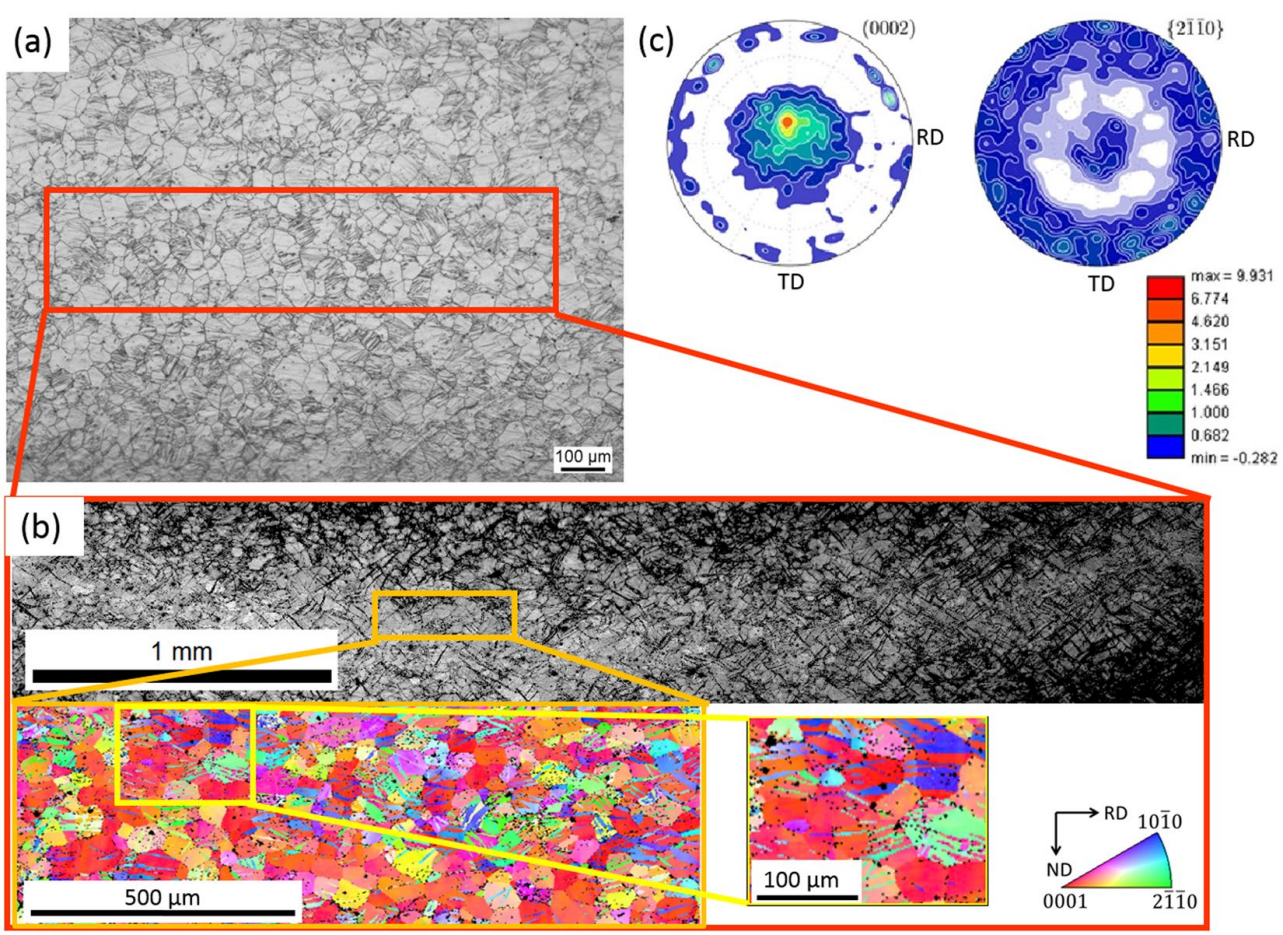
Microstructure (optical microscopy (**a**), electron backscatter diffraction (EBSD) (**b**), x-ray diffraction (**c**)) of the new Mg-Al-Ca alloy revealing grain sizes of about 35-50 µm and a prevalent basal texture with a slight TD texture component. (**a**) Optical micrograph of a sample deformed to 10% engineering strain, (**b**) secondary electron micrograph and EBSD map after deformation to 10% engineering strain, (**c**) Pole figure plots of an undeformed sample.

**Figure 5:**
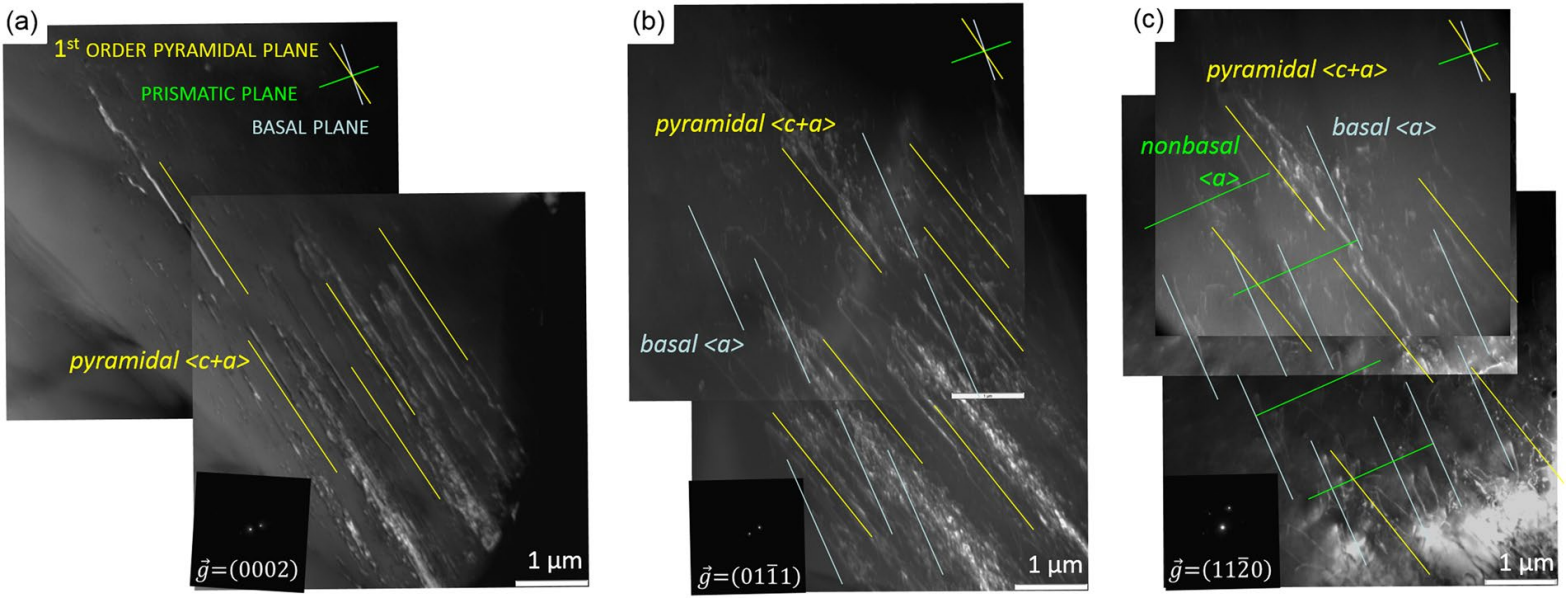
TEM weak beam dark field micrographs after deformation to 2% engineering strain taken under different diffraction conditions prove the predominant activity of pyramidal <c + a> and basal <a> dislocation slip. Small capitals mark planes and italic letters mark slip systems.

To unravel the reasons for the observed ductility increase of the new Mg-Al-Ca alloy and prove if it is caused by increased activity of non-basal dislocation slip and a decreased I_1_ SFE, as predicted by *ab initio*, we have performed microstructure characterization, Figs 4 and 5. These investigations confirm that the observed ductility increase is not caused by texture engineering, nano-structuring, grain size reduction, twinning activation or second phase dispersions but, indeed, simply by a solid solution effect facilitating the activation of non-basal <c + a> dislocation slip, Fig. 5. $\{10\bar{1}2\}\langle 10\bar{1}\bar{1}\rangle$ tensile deformation twinning was observed as one of the predominant deformation systems in pure Mg and most Mg alloys – together with basal <a> dislocation slip. However, basal <a> slip and tensile twinning alone offer only 3 independent deformation systems and, hence, do not fulfil the von Mises criterion requiring at least 5 independent deformation systems for compatible polycrystalline deformation.

Optical microscopy, scanning electron microscopy (SEM), electron backscatter diffraction (EBSD) and transmission electron microscopy (TEM) show that the new Mg-1Al-0.1Ca alloy is a single-phase solid solution alloy without any second phase precipitates, Fig. 4. The material has a grain size of 35–50 µm and a prevalent basal texture with a slight TD texture component, hence, it is microstructurally comparable to the reference alloys containing Y and RE elements given in Fig. 3 ^6, 13^. As evident from the electron backscatter diffraction (EBSD) map in Fig. 4b, significant tensile twinning took place during deformation of the new Mg-Al-Ca alloy. No compression twinning was observed. We find that most grains contain tensile twins, preferentially of one variants, however, grains with more than one activated twin variant are also present, Fig. 4b. The occurrence of twins and twin volume fraction (~40% after 10% deformation) are similar as has been observed for pure Mg and Mg alloys. No additional twinning systems, such as $\{10\overline{1}1\} < 10\overline{1}2 >$ compression and $\{10\overline{1}1\}\{10\overline{1}2\}$ secondary deformation twinning, were activated, which would have increased the number of independent deformation systems. We therefore propose that the activity of deformation twinning is not significantly changed through the addition of 1 wt.-% Al and 0.1 wt.-% Ca in solid solution and, thus, the only mechanism remaining to explain the observed ductility is the increased activity of non-basal dislocation slip.

TEM analysis of the active dislocations and slip systems using the $\overrightarrow{g}\cdot \overrightarrow{b}$ criterion, where $\overrightarrow{g}$ is the diffraction vector and $\overrightarrow{b}$ is the Burgers vector, clearly reveals activity of non-basal <c + a> and basal <a> dislocation slip, Fig. 5. According to the $\overrightarrow{g}\cdot \overrightarrow{b}$ criterion only dislocations with a < c > -component are visible under $\overrightarrow{g}=(0002)$ conditions (a); only dislocations with an <a> -component are visible under $\overrightarrow{g}=(11\bar{2}0)$ conditions (c); and both, <a> and <c> components, are visible under $\overrightarrow{g}=(01\bar{1}1)$ conditions (b) in Fig. 5. The plane traces as marked in Fig. 5 unambiguously identify the glide plane of the <c + a> dislocation being the $(10\bar{1}1)$ 1^st^ order pyramidal plane confirming them being not dissociated and relaxed onto the basal plane. This pronounced activity of basal <a> and pyramidal <c + a> dislocations enables the increased ductility in the as-homogenized and un-engineered state observed for the new Mg-1Al-0.1Ca alloy.

The TEM micrographs shown in Fig. 5 are typical images of this alloy in which several different grains were examined. When comparing the relative amounts of <c + a> dislocations and <a> dislocations of this Mg-Al-Ca alloy with Mg-Y and Mg-RE alloys^12, 13, 64^ the ratio $< {\rm{c}}+{\rm{a}} > {\rm{dislocations}}/ < {\rm{a}} > {\rm{dislocations}}$ is slightly lower in the Mg-Al-Ca alloy, in agreement to the computed YSI values. However, TEM does not provide significant statistical relevance due to the small volumes investigated and EBSD does only give the number of geometrically necessary dislocations. Therefore a sound quantitative comparison is not possible using electron microscopy.

**Figure 6:**
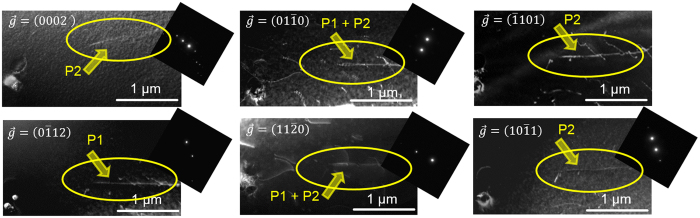
TEM weak beam dark field micrographs of an I_1_ intrinsic stacking fault (I_1_ SF) in the new Mg-Al-Ca alloy taken under different diffraction conditions. The visibility of the two bounding partial dislocations is indicated by P1 (partial dislocation 1) and P2 (partial dislocation 2), respectively. The corresponding dissociation width amounts to 18 nm.

In line with the previous studies on Mg-Y and Mg-RE alloys which have revealed a correlation of the increased activity of <c + a> dislocation slip with the I_1_ SFE further TEM analysis of the I_1_ SFE was performed on undeformed material. By using a set of six different diffraction vectors $\overrightarrow{g}$ from two different zone axes the presence of I_1_ SFs with a dissociation width of 18 ± 3 nm was observed in the new Mg-Al-Ca alloy, Fig. 6. A dissociation width of 18 ± 3 nm corresponds to an I_1_ SFE of 2.5 ± 0.5 mJ/m²^12, 13^, being significantly lower than in pure Mg (20 mJ/m², corresponding to a dissociation width <2 nm)^12, 13^.

In conclusion, we have designed a new RE-free ductile Mg alloy by using a computer assisted rapid alloy assessment through an *ab initio* derived non-linear relation of fundamental atomic parameters. The new alloy contains Al and Ca in dilute solid solution, two inexpensive and non-toxic elements. This, by metallurgical standards, tiny doping with Al and Ca leads to a huge macroscopic effect by activating in addition to basal also pyramidal slip. Contrary to most alloy concepts, our *ab initio* based strategy suggested to reduce doping levels, which can be regarded as a ‘less-is-more’ alloying principle. We also emphasize that the alloy does not require any additional measures such as texture engineering, nano-structuring, grain size reduction, activation of twinning systems other than $\{10\bar{1}2\}\langle 10\bar{1}\bar{1}\rangle$ tensile twinning or second phase dispersions. Future research directions should focus on additional microstructure and texture engineering of this alloy class, for example by the addition of further alloying elements to form strengthening precipitates (not being Mg_2_Ca Laves phase), texture modification and grain refinement.

## Experimental Methods

The alloys were prepared from pure Mg, pure Ca and pure Al (all having a purity of >99,98%) in an induction furnace under Ar pressure. To homogenize the microstructure and remove elemental segregations, the as-cast block was hot rolled to 50% thickness reduction at 430 °C and recrystallization annealed at 450 °C for 15 min followed by water quenching.

Samples for optical microscopy and electron backscatter diffraction (EBSD) analysis were prepared by mechanical grinding and polishing followed by electrolytical polishing using the electrolyte AC2 (Struers). Transmission electron microscopy (TEM) samples were prepared from 3 mm discs by mechanical grinding and twin-jet polishing until perforation using a solution of 3% perchloric acid in ethanol as electrolyte.

Tensile testing was done at room temperature and an initial strain rate of 10^−4^ s^−1^ using an electromechanical testing machine (DZM) with an accuracy of 0.17 MPa. Texture measurements were performed on a Bruker D8 Advance x-ray diffraction instrument. EBSD measurements were conducted using a Zeiss FIB XB1540 SEM and TEM observation were performed on a Philips CM20 TEM.
